# A rescue technique for failed stent deployment by the stent-in-stent technique using mesh broken by holmium laser ablation

**DOI:** 10.1055/a-2723-1543

**Published:** 2025-11-04

**Authors:** Takeshi Ogura, Jun Matsuno, Takafumi Kanadani, Junichi Nakamura, Hiroki Nishikawa

**Affiliations:** 1 Pancreatobiliary Advanced Medical Center, Osaka Medical and Pharmaceutical University Hospital, Osaka, Japan; 2 13010 Endoscopy Center, Osaka Medical and Pharmaceutical University, Osaka, Japan; 3 2nd Department of Internal Medicine, Osaka Medical and Pharmaceutical University, Osaka, Japan

Uncovered self-expandable metal stent (UCSEMS) deployment by the stent-in-stent (SIS) technique can be attempted in patients with unresectable malignant hilar biliary obstruction (MHBO) 1 2 . However, due to improvements in systemic chemotherapy, longer stent patency is required. Compared with the UCSEMS, the covered SEMS (CSEMS) may have benefits, such as prolonged stent patency, but if CSEMS deployment is performed for MHBO, bile duct branch obstruction can occur as a complication. To overcome this potential complication, a multi-hole SEMS (MHCSEMS; HANAROSTENT Biliary Multi-hole Benefit; M.I. Tech Co., Ltd, Pyeongtaek, South Korea) has become available. This stent prevents stent migration via small tissue ingrowths that form in the multiple small (1.8-mm) side holes along the covering membrane. However, during SIS, guidewire passage into the lateral side after SEMS deployment may sometimes be challenging. A rescue technique for failed stent deployment by the SIS technique using mesh broken by holmium laser ablation is described.

First, after successful guidewire deployment into the right and left hepatic bile ducts, hilar obstruction was observed on cholangiography ( Fig. 1 ). Next, the MHCSEMS was successfully deployed into the right hepatic bile duct. Guidewire insertion into the left bile duct through the mesh of the MHCSEMS was attempted, but failed. Cholangioscope insertion into the biliary tract was attempted. The orifice of the left hepatic bile duct was successfully identified by the landmark guidewire ( Fig. 2 ). Then, the mesh and covering membrane were broken by holmium laser ablation ( Fig. 3 ). After this procedure, guidewire insertion into the left bile duct was successfully performed ( Fig. 4 ). Finally, MHCSEMS deployment into the left bile duct was successfully performed without any adverse events ( Fig. 5 ; Video 1 ).

**Fig. 1:**
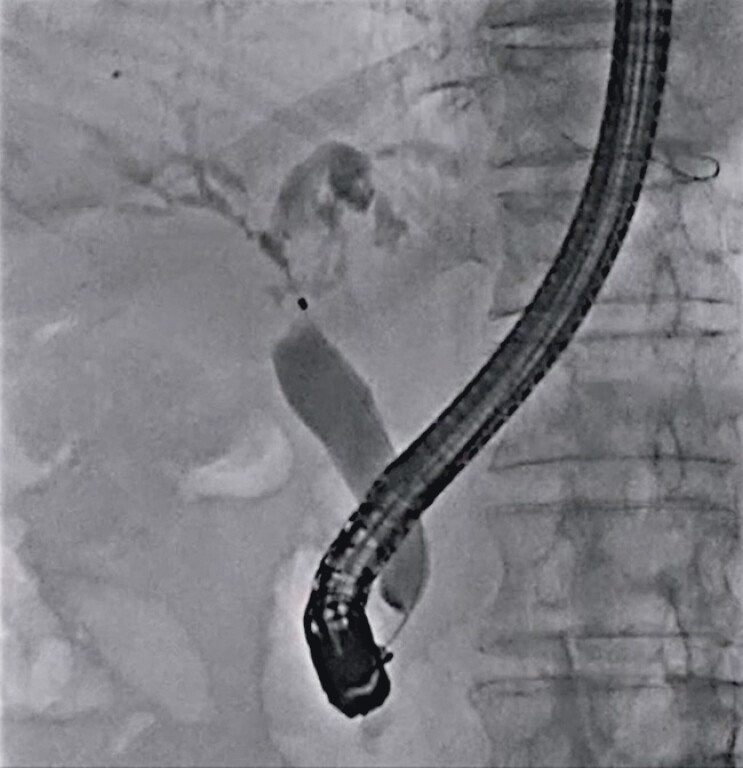
Hilar obstruction is observed on cholangiography.

**Fig. 2:**
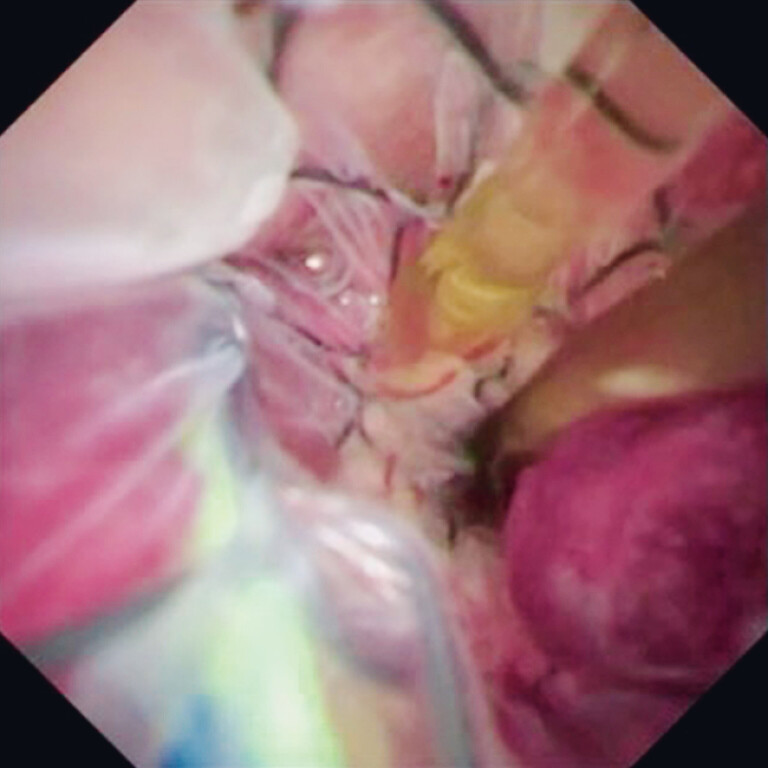
The orifice of the left hepatic bile duct is successfully identified by the landmark guidewire.

**Fig. 3:**
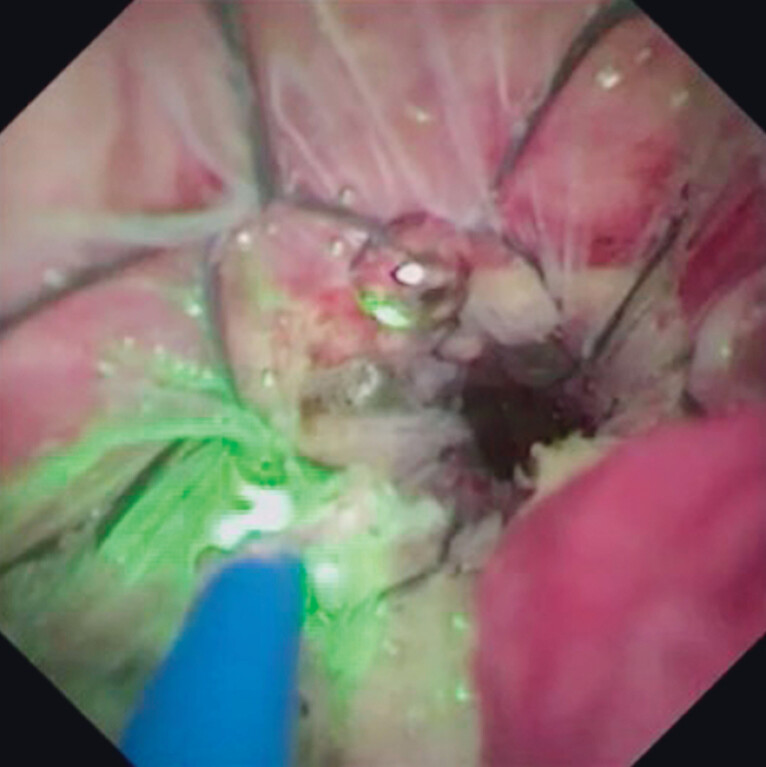
The mesh and covering membrane are broken by holmium laser ablation.

**Fig. 4:**
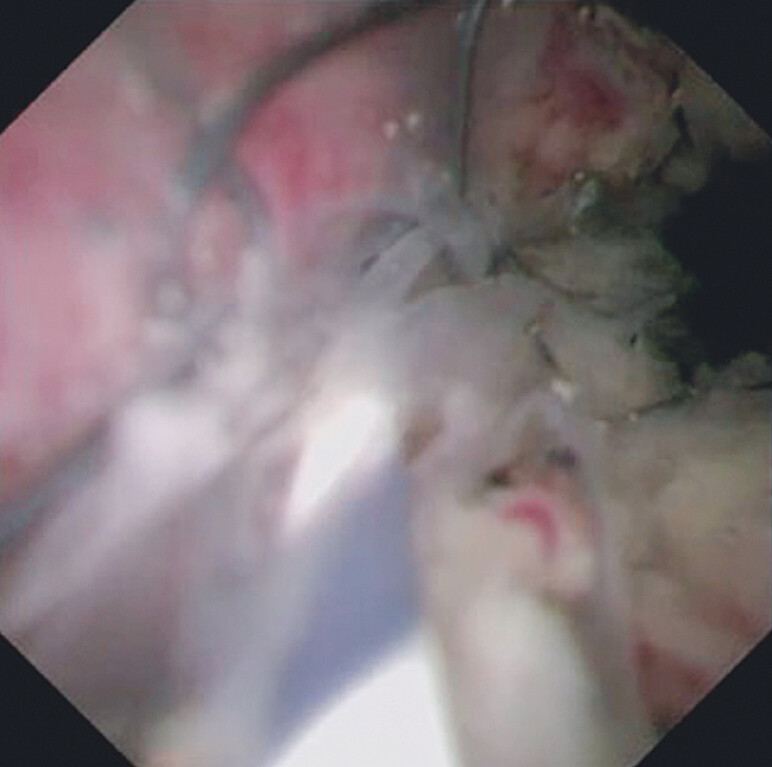
Guidewire insertion into the left bile duct is successfully performed.

**Fig. 5:**
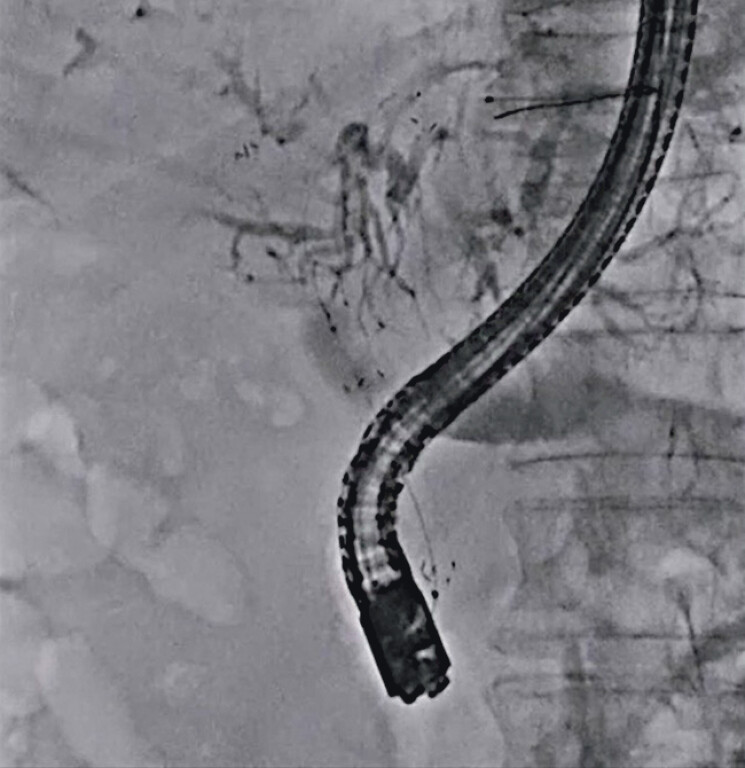
Stent deployment into the left bile duct is successfully performed.

The mesh and covering membrane are broken by holmium laser ablation.Video 1

In conclusion, the technique presented might be helpful as a rescue technique in cases of the failed SIS technique.

Endoscopy_UCTN_Code_TTT_1AR_2AZ

## Notes

Endoscopy E-Videos https://eref.thieme.de/e-videos
*E-Videos* is an open access online section of the journal *Endoscopy* , reporting on interesting cases and new techniques in gastroenterological endoscopy. All papers include a high-quality video and are published with a Creative Commons CC-BY license. Endoscopy E-Videos qualify for HINARI discounts and waivers and eligibility is automatically checked during the submission process. We grant 100% waivers to articles whose corresponding authors are based in Group A countries and 50% waivers to those who are based in Group B countries as classified by Research4Life (see: https://www.research4life.org/access/eligibility/ ). This section has its own submission website at https://mc.manuscriptcentral.com/e-videos .
